# Targeted 3.34-Million CpG Site Sequencing Reveals Preliminary Cord Blood Epigenetic Alterations of Autism Spectrum Disorder: A Pilot Study Highlighting the *PCDHA1*–*PCDHA8* Cluster

**DOI:** 10.3390/ijms27188038

**Published:** 2026-09-09

**Authors:** Marina Armas-González, Nieves Luisa González-González, Enrique González-Dávila, Olivia Orribo-Morales, José Ramón Castro-Conde, Candelaria González-Campos, Carlos Flores, José Miguel Lorenzo-Salazar, Rafaela González-Montelongo, Adrián Muñoz-Barrera, Erika Padrón-Pérez, Laura Tascón-Padrón, María Núñez Catalán

**Affiliations:** 1 Obstetrics and Gynecology Department, Sección de Medicina, Facultad de Ciencias de la Salud, University of La Laguna (ULL), 38320 La Laguna, Spain; marmasglez@gmail.com (M.A.-G.); oorribomorales@gmail.com (O.O.-M.); jcastro@ull.edu.es (J.R.C.-C.); lalytfe@gmail.com (C.G.-C.); erikapadronperez@gmail.com (E.P.-P.); marianuzcan@gmail.com (M.N.C.); 2 Department of Child and Adolescent Psychiatry, Hospital General Universitario Gregorio Marañón, 28007 Madrid, Spain; 3 Mathematics, Statistics and Operations Research Department (IMAULL), University of La Laguna (ULL), 38320 La Laguna, Spain; 4 Department of Obstetrics and Gynecology, Canary Islands University Hospital, 38320 La Laguna, Spain; 5 Pediatrics Department, Canary Islands University Hospital, 38320 La Laguna, Spain; 6 Research Unit, Hospital Universitario Ntra. Sra. de Candelaria, Instituto de Investigación Sanitaria de Canarias (IISC), 38010 Santa Cruz de Tenerife, Spain; cflores@ull.edu.es; 7 CIBER de Enfermedades Respiratorias (CIBERES), Instituto de Salud Carlos III, 28029 Madrid, Spain; 8 Genomics Division, Instituto Tecnológico y de Energías Renovables (ITER), 38600 Santa Cruz de Tenerife, Spain; jlorenzo@iter.es (J.M.L.-S.); rgonzalezmontelongo@iter.es (R.G.-M.); amunoz@iter.es (A.M.-B.); 9 Department of Obstetrics and Prenatal Medicine, University Hospital Bonn, 53127 Bonn, Germany; l.tasconpadron@gmail.com

**Keywords:** autism, epigenetic, DNA, cord blood, neurodevelopment, maternal obesity, diabetes, enrichment analysis, pregnancy, protocadherins

## Abstract

Rising reported Autism Spectrum Disorder (ASD) prevalence underscores the need to explore early perinatal biological markers. To conduct an exploratory pilot investigation of cord blood DNA methylation patterns as potential early epigenetic candidate loci associated with ASD in infants born to mothers with obesity and gestational diabetes. This prospective pilot study analyzed 14 mother-infant pairs comprising infants born to mothers with obesity and gestational diabetes later diagnosed with ASD (*n* = 2); healthy infants born to mothers with obesity and diabetes (IODM, *n* = 6); and healthy controls (IHM, *n* = 6). Targeted methyl-capture sequencing (covering 3.34 million CpG sites) identified differentially methylated region (DMR)-associated genes. Neurodevelopment was evaluated at 24–26 months using Bayley-III scales. ASD infants showed lower Bayley-III scores across all domains. In this small cohort (*n* = 2 ASD cases), we identified 249 DMR-associated genes common to both ASD vs. IHM and ASD vs. IODM comparisons, representing preliminary candidate loci. Enrichment analysis identified homophilic cell–cell adhesion as the most significant pathway (fold enrichment = 8.28; FDR = 6.31 × 10^−6^), driven by the *PCDH1*–*PCDHA8* cluster, alongside morphogenesis in neuron differentiation (fold enrichment = 3.45; FDR = 2.08 × 10^−2^). Although prospective validation in larger independent cohorts is required, these pilot results suggest that cord blood *PCDHA1*–*PCDHA8* cluster methylation alterations represent preliminary candidate loci for hypothesis generation and future biomarker evaluation, regardless of maternal metabolic status.

## 1. Introduction

Autism spectrum disorder (ASD) is defined according to the Diagnostic and Statistical Manual of Mental Disorders (DSM-5) criteria, providing a standardized clinical framework for evaluation [1]. Additionally, recent advances have further elucidated the complex molecular and genetic architecture of ASD, emphasizing key biological pathways disrupted in the condition [2]. According to the World Health Organization, ASD is characterized by a degree of difficulty with social interaction and communication, along with atypical patterns of behavior and activity [3]. The reported prevalence of ASD has shown a marked increase in recent decades, a trend that partly reflects changes in diagnostic criteria, awareness, and ascertainment (1 in 36 children in the USA, 2020) [4].

Among the risk factors clearly associated with ASD are those affecting the intrauterine environment, especially maternal obesity and gestational diabetes [5,6,7]. These medical conditions are the most frequent complications of pregnancy, and their prevalence has increased exponentially in recent years [8]. Although this epidemiological parallel with the rise in ASD diagnoses does not in itself demonstrate a mechanistic relationship, shared risk factors and potential environmental mediators—including epigenetic alterations in fetal DNA—have been proposed as plausible links.

Multiple studies have documented epigenetic alterations in the peripheral blood DNA of children and adults diagnosed with ASD, as well as in post-mortem brain tissue from both humans and animal models [9]. Concurrently, it has been proposed that epigenetic signatures identifiable at birth could serve as early ASD biomarkers. Such early detection is crucial, as behavioral symptoms often remain latent until the disorder is well established, frequently delaying clinical diagnosis until 3–4 years of age, or even later [10]. Most of these studies have been restricted to candidate genes, and those analyzing the whole epigenome have fundamentally employed microarray techniques with constrained CpG coverage; consequently, the reproducibility of findings across cohorts remains limited [11].

In this pilot study, we aimed to generate working hypotheses by characterizing the cord blood methylome of newborns who later developed ASD using a sequencing-based methyl-capture approach—offering fourfold the coverage of standard microarrays.

## 2. Results

### 2.1. Maternal Characteristics and Perinatal Outcomes and Neurodevelopment

Maternal characteristics: Mothers in the ASD group were significantly older (38.5 ± 2.1 vs. 33.2 ± 1.7 years; *p* = 0.011) and had a higher BMI (43.5 ± 4.4 vs. 20.0 ± 1.9 kg/m^2^; *p* < 0.001) than the healthy control group (IHM). However, no statistically significant differences were found when comparing the ASD group to the IODM group in terms of age (*p* = 0.158), BMI (*p* = 0.120), or parity (*p* = 0.643).

Perinatal outcomes: No significant differences were observed between the ASD group and both control groups (IODM and IHM) (gestational age, birth weight, or Apgar scores) (Table S1).

**Figure 1:**
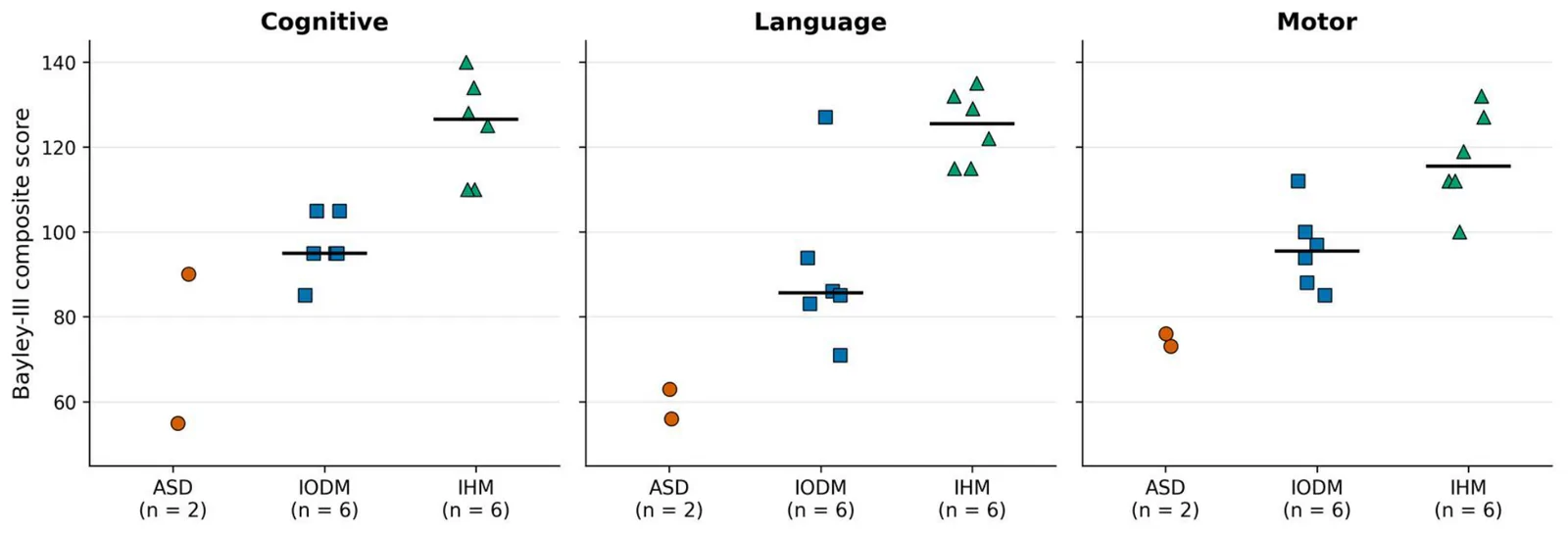
Bayley-III test domain scores for the cognitive, language, and motor domains in infants with ASD (orange circles, *n* = 2) and in the two control groups, infants of mothers with obesity and gestational diabetes (IODM, blue squares, *n* = 6) and healthy infants born to healthy mothers (IHM, green triangles, *n* = 6). Individual data points are shown for all participants; horizontal black lines indicate group medians for the IODM and IHM groups.

Bayley-III neurodevelopmental scores: Infants with ASD scored significantly lower than both the IODM and IHM groups across all domains: Cognitive (*p* = 0.050 and *p* = 0.006, respectively), Language (*p* = 0.010 and *p* < 0.001), and Motor (*p* = 0.024 and *p* = 0.003) (Figure 1 and Table S1).

### 2.2. Methylation Analysis

#### 2.2.1. DMR-Associated Genes Common to Both Comparisons (ASD vs. IHM and vs. IODM)

Of the 3.34 million CpG sites analyzed per infant, epigenetic analysis identified substantial methylation differences in both comparisons. In the ASD vs. IHM comparison, 10,414 DM CpGs were detected, mapping to 576 DMRs overlapping 516 unique genes. In the ASD vs. IODM comparison, 11,202 DM CpGs were identified, associated with 750 DMRs located within 679 unique genes. In both comparisons, hypomethylated CpGs were slightly more prevalent than hypermethylated ones (58.5% and 57.5%, respectively).

Notably, 249 DMR-associated genes were common to both comparisons, representing 48.3% of genes identified in the ASD vs. IHM comparison and 36.7% of those in the ASD vs. IODM comparison (Table S2). This overlapping subset represents an exploratory set of candidate genes that show altered cord blood DNA methylation in these two ASD cases regardless of whether controls were born to healthy or metabolically impaired mothers.

#### 2.2.2. Functional Enrichment

**Figure 2:**
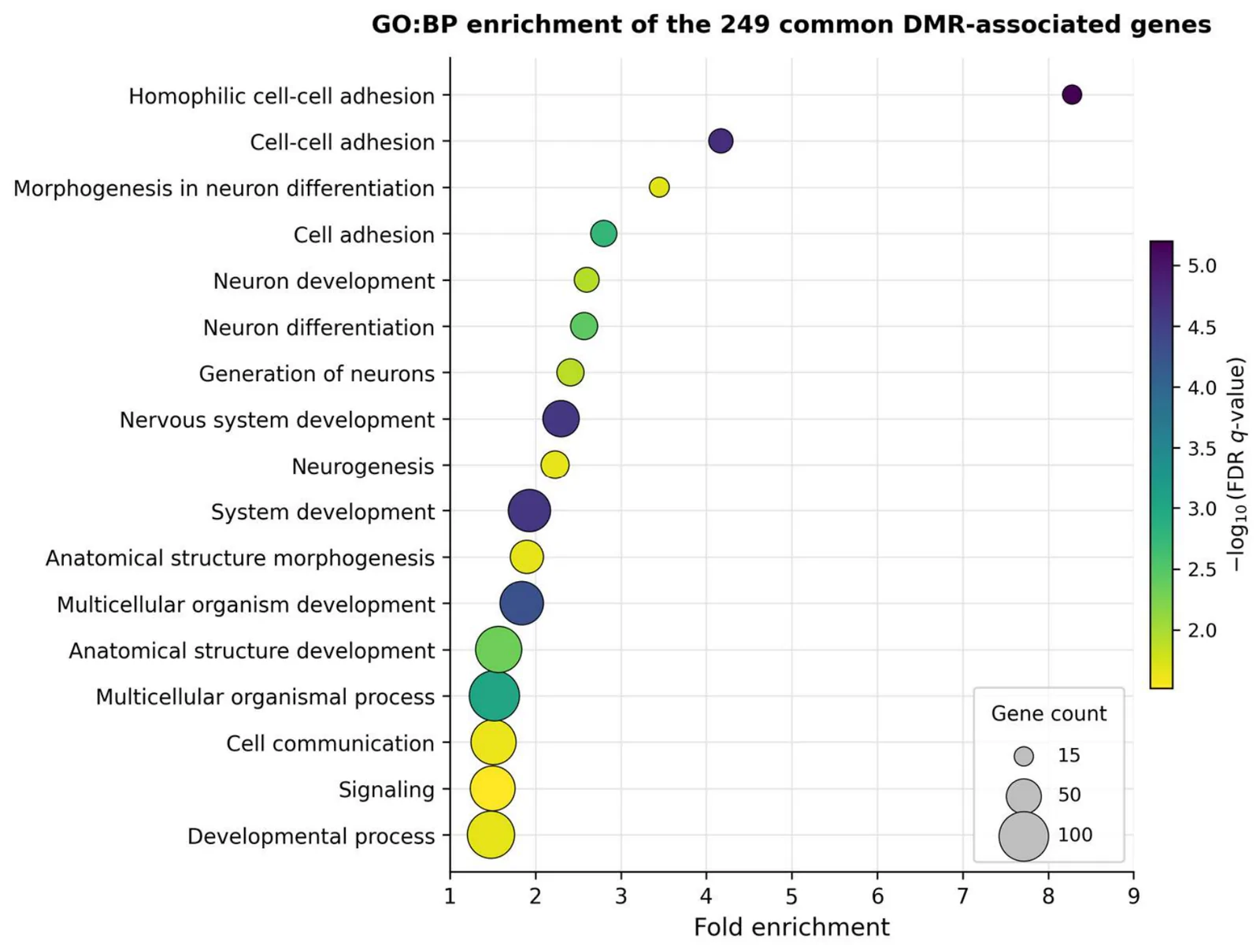
Dot plot of the significantly enriched Gene Ontology Biological Process (GO:BP) terms identified for the 249 DMR-associated genes common to both comparisons (ASD vs. IODM and ASD vs. IHM). The x-axis shows the fold enrichment of each term; dot size is proportional to the number of DMR-associated genes annotated to the term, and color indicates statistical significance (−log10 of the FDR-corrected q-value). Terms are ordered by fold enrichment (numerical values in Table S3).

Gene Ontology [12] enrichment analysis of the 249 common DMR-associated genes revealed a consistent neurodevelopmental signature organized around four converging biological pathways related to cell adhesion and neural differentiation. The most significantly enriched pathway was homophilic cell–cell adhesion (fold enrichment = 8.28; FDR = 6.31 × 10^−6^), driven primarily by the *PCDH1*–*PCDHA8* cluster. Additional significantly enriched pathways included morphogenesis in neuron differentiation (fold enrichment = 3.45; FDR = 2.08 × 10^−2^; including its sub-branches: neuron development, developmental process, anatomical structure development, neuron differentiation, generation of neurons, neurogenesis, nervous system development, system development, multicellular organism development, multicellular process, and anatomical structure morphogenesis), cell communication (fold enrichment = 1.51; FDR = 2.38 × 10^−2^), and signaling (fold enrichment = 1.50; FDR = 3.03 × 10^−2^) (Table S3 and Figure 2).

#### 2.2.3. Overlap with the SFARI Gene Database

Among the 249 common DMR-associated genes, 29 overlapped with established ASD-risk genes cataloged in the SFARI database [13]. Three genes were classified as Category 1 (High Confidence): *GABRB3*, *MYT1L*, and *PCDH19*. The largest group (*n* = 20) was classified as Category 2 (Strong Candidate), featuring the *PCDHA* gene cluster (*PCDHA1*–*PCDHA8*), *KCNQ2*, *CNTN4*, and other key synaptic and cell-adhesion molecules (Table 1 and Table S4).

### 2.3. Comparison Between ASD vs. IHM

#### 2.3.1. Functional Enrichment

Gene Ontology [12] enrichment analysis of DMR-associated genes identified in this comparison revealed 10 significantly enriched pathways. Notably, five of these pathways were associated with neurodevelopmental and neurological functions, specifically: Positive regulation of excitatory postsynaptic potential (fold enrichment = 8.72; FDR = 0.0304), axon guidance (fold enrichment = 3.10; FDR = 0.0474), homophilic cell-to-cell adhesion (fold enrichment = 4.93; FDR < 0.0001), chemical synaptic transmission (fold enrichment = 2.49; FDR = 0.0426), and central nervous system development (fold enrichment = 1.92; FDR = 0.0358). Table S5 presents the corresponding results, while Table S6 displays the complete enrichment analysis.

#### 2.3.2. Overlap with the SFARI Gene Database

The integration of identified DMR-associated genes with the SFARI Gene database [13] revealed that 45 genes were common to both comparisons. Notably, 8 genes were identified as Category 1 (High Confidence) risk factors, including *CACNA1A*, *CASZ1*, *GABRB3*, *GRIN1*, *MYT1L*, *NLGN2*, *PCDH19*, and *ZMYND8*. The largest proportion of overlapping genes (*n* = 34) was classified as Category 2 (Strong Candidate), featuring essential synaptic and cell-adhesion molecules such as *KCNQ2*, *CNTN4*, and the *PCDHA1*–*PCDHA8* cluster (Table 1 and Table S4).

### 2.4. Comparison Between ASD vs. IODM

#### 2.4.1. Enrichment Analysis

Functional enrichment analysis of DMR-associated genes identified in the ASD versus IODM comparison showed four significantly enriched biological processes: homophilic cell–cell adhesion, neuron development, multicellular organism development, and multicellular organism process (Table S7).

#### 2.4.2. Overlap with the SFARI Gene Database

Cross-referencing our detected DMR-associated genes with the SFARI database [13] uncovered a substantial intersection with 52 well-characterized ASD-risk loci. Seven genes were identified as Category 1 (High Confidence) risk factors, including *ANKRD11*, *DEAF1*, *DMPK*, *EBF3*, *GABRB3*, *MYT1L*, and *PCDH19*. The largest group (*n* = 36) was classified as Category 2 (Strong Candidate), featuring essential neurodevelopmental genes including the *PCDHA1*–*PCDHA10* cluster (Table 1 and Table S4).

## 3. Discussion

This is a hypothesis-generating pilot study utilizing a high-breadth sequencing approach to characterize the cord blood methylome in infants with ASD, achieving a fourfold increase in genomic coverage compared to traditional microarray platforms. The principal finding is the identification of an exploratory candidate gene set comprising 249 DMR-associated genes that showed consistent methylation differences in ASD infants relative to both metabolically exposed (IODM) and healthy (IHM) control groups. Notably, this candidate gene set included a cluster of protocadherin genes (*PCDHA1*–*PCDHA8*). While genetic variants in this cluster have been previously linked to ASD, epigenetic alterations in these loci have not been reported to date. The convergence of these findings across both comparisons suggests, at a strictly hypothesis-generating level, that these epigenetic alterations may be associated with ASD itself rather than exclusively with the intrauterine environment conditioned by maternal obesity and gestational diabetes; nevertheless, with only two ASD cases, an ASD-specific signature cannot be established, and this interpretation requires confirmation in larger independent cohorts.

The protocadherin alpha gene cluster *PCDHA1*–*PCDHA8* represents a specific subset of variable exons within the protocadherin alpha cluster. These specific isoforms play critical roles in establishing neuronal identity and neural circuit assembly [14]. Furthermore, *PCDH19*—another member of the same family located on the X chromosome and frequently linked to ASD in both genetic [15] and epigenetic [9] —also exhibited the identical DMR across both comparisons in our study.

The consistent enrichment of neurogenesis, neuron differentiation, and nervous system development pathways within the overlapping gene set from both comparisons reflects a potential convergent disruption of early neurodevelopmental programming—a pattern compatible with the prenatal timing of the epigenetic signal captured in cord blood. The additional pathways uniquely enriched in the ASD vs. IHM comparison—axon guidance, chemical synaptic transmission, and positive regulation of excitatory postsynaptic potential—extend this neurodevelopmental narrative to include processes governing synaptic connectivity and functional balance.

Regarding the scope of the functional analysis, enrichment was intentionally restricted to the GO Biological Process (GO:BP) ontology. GO:BP terms integrate multi-protein biological cascades and developmental cellular programs (e.g., neurogenesis, axon guidance, synaptic transmission) that map directly onto the neurodevelopmental hypotheses addressed in this study, whereas the Cellular Component (GO:CC) and Molecular Function (GO:MF) ontologies primarily categorize static subcellular localization and elemental binding activities, which provide broader but less specific insight in exploratory epigenome-wide analyses. Restricting the analysis to a single ontology also limited the number of statistical tests performed in this small pilot cohort.

The overlap of 29 common genes with the SFARI Gene database provides preliminary biological validation for our findings, despite the limitations of a small sample size. Notably, the three high-confidence (Category 1) shared SFARI genes—*GABRB3*, *MYT1L*, and the aforementioned *PCDH19*—represent well-established ASD-risk loci with distinct biological roles. *GABRB3* encodes the beta3 subunit of the *GABA* receptor and is critical for inhibitory synaptic transmission, with mutations linked to both ASD and epileptic encephalopathy [16,17]. *MYT1L* is a transcription factor expressed predominantly in neurons and is heavily associated with intellectual disability and ASD [18].

Our identification of five X-linked DMR-associated genes—*AMER1*, *ARHGAP6*, *DOCK11*, *PCDH19*, and *ZIC3*—that intersect with validated ASD signatures from Mordaunt et al. [19] is highly consistent with the known sex bias in ASD and the established enrichment of ASD-associated DMRs on the X chromosome.

Two genes—*CNTNAP2* and the previously highlighted *MYT1L*, both of which exhibited associated DMRs across both comparisons in our study—were replicated in females across two independent cohorts by Mouat et al. [20] using whole-genome bisulfite sequencing and methyl-capture approaches similar to our own. Furthermore, our analysis identified differential methylation in two members of the *GALNT* family, *GALNT2* and *GALNT9*, in the ASD versus IHM and ASD versus IODM, respectively, whereas Mouat et al. [20] reported methylation differences specifically in *GALNT2*. These genes are highly enriched in the developing human brain and regulate the glycosylation of cell-surface receptors essential for axonal guidance and synapse formation [21].

In a prior cord blood epigenome study of newborns exposed to maternal type 1 diabetes, González-González et al. [22] demonstrated that during neurodevelopmental evaluations conducted at two years of age, methylation levels at the *MYT1L*-associated differentially methylated region (DMR) correlated with Bayley-III language domain scores, whereas those at *CNTNAP2* correlated with motor domain scores. In a subsequent study evaluating 30 mothers with gestational diabetes and their infants against 30 healthy controls, the same authors [23] observed that cognitive domain scores significantly correlated with methylation levels at DMRs associated with the protocadherin gamma gene cluster (*PCDHGA1*–*PCDHGA8*), further underscoring the clinical relevance of these epigenetic alterations.

However, several systematic reviews have noted a limited replication of specific differentially methylated (DM) candidate genes across independent ASD studies encompassing newborns, infants, adults, and postmortem tissues [9,11]. Dall’Aglio et al. [24] found that only a few markers, notably *OR2L13*, showed consistent results across cohorts in adults with ASD. Stoccoro et al. [25] identified a distinct pediatric ASD signature involving *BCL2*, *RELN*, and *OR2L13*. Our analyses similarly detected differential methylation at *OR2L13* in newborns across both comparisons. Supporting these findings, similar epigenetic alterations have been documented across analogous maternal metabolic contexts; Howe et al. [26] reported methylation differences in *OR2L13* in neonates exposed to maternal gestational diabetes mellitus (GDM), and similarly, Gonzalez-Gonzalez et al. [22,23] observed these epigenetic alterations in newborns exposed to both gestational and maternal type 1 diabetes.

From a methodological perspective, this pilot study presents two key advantages: the use of targeted methyl-capture sequencing covering 3.34 million CpG sites—offering fourfold the coverage of standard microarrays—and a dual-control design. By comparing ASD infants against both healthy controls (IHM) and metabolically matched non-ASD controls (IODM), this approach allows us to differentiate potential ASD-specific epigenetic signatures from baseline fetal alterations induced by the intrauterine metabolic environment conditioned by maternal obesity and diabetes.

The most critical limitation of our study is its sample size; the ASD group comprised only two infants, which severely restricts statistical power. With only two ASD cases, differential methylation estimates are inherently unstable: they are highly sensitive to individual-level biological and technical variability, sampling variance is large, and effect sizes may be inflated by a winner’s-curse phenomenon. Logistic regression models fitted to such small groups can yield wide confidence intervals, and the reported q-values and fold enrichments should therefore be interpreted as preliminary screening signals rather than robust effect estimates. A constraint of our small sample size was the inability to adjust for additional clinical and environmental confounding factors, including maternal glycemic control throughout pregnancy, as well as socioeconomic or parental educational status. Consequently, all results must be interpreted as strictly hypothesis-generating. Although the prospective design, careful matching, and use of two complementary control groups partially offset this constraint, replication in larger independent cohorts alongside transcriptomic or proteomic evaluations is crucial prior to clinical application.

The potential limitation of using cord blood as a surrogate to reflect neural tissue behavior is effectively resolved by a growing body of evidence demonstrating blood-brain methylation concordance. Hannon et al. [27] demonstrated that inter-individual variation at specific genomic regions is highly correlated between blood and brain tissue. More recently, Mouat et al. [20] showed that newborn blood DMRs overlap substantially with cord blood, peripheral blood, and placental signatures—though not with post-mortem adult cortex—suggesting that cord blood captures relevant prenatal epigenetic programming events.

## 4. Materials and Methods

We conducted a prospective, case–control pilot study (1:3 ratio) involving a discovery cohort of 14 mother-infant pairs. Participants were recruited between June 2021 and January 2024 from a larger longitudinal study at the University Hospital of the Canary Islands (Tenerife, Spain), which originally enrolled 40 mothers with diabetes and 40 healthy controls, with offspring followed for 24–36 months. Of the initial 80 enrolled pairs, 28 were excluded due to obstetric complications, birth weight below the 10th percentile, gestational age < 37 weeks, or loss to pediatric follow-up. Among the remaining eligible offspring followed through 24–26 months, 2 infants in the diabetic/obese cohort received a formal clinical diagnosis of ASD. From the remaining normotypical offspring, 6 infants of mothers with obesity and diabetes (IODM) and 6 infants of healthy mothers (IHM) were selected as matched controls based on sex, mode of delivery, and ethnicity (Supplementary Figure S1).

The current study cohort was stratified into three groups, two of which are non-ASD control groups: (1) infants born to mothers with obesity and diabetes later diagnosed with ASD (*n* = 2); (2) infants born to mothers with obesity and diabetes (IODM group; *n* = 6); and (3) healthy infants born to healthy mothers (IHM group; *n* = 6).

This study was conducted in accordance with the Declaration of Helsinki and was approved by the Clinical Ethics Committee of the Canary Islands University Hospital Complex, Spain (CHUC-201817), approved on 28 March 2019, and written informed consent was obtained from all mothers prior to participation.

Inclusion criteria included singleton pregnancy, delivery via vaginal or elective cesarean section under regional anesthesia, and gestational age > 37 weeks. Participants were required to be free of maternal pathologies, except for the IODM group, which specifically included mothers with obesity and diabetes.

We strictly excluded cases involving medical or obstetric complications, smoking or substance abuse, or medication other than insulin. Exclusion criteria also included a birthweight below the 10th percentile according to customized Spanish growth curves [28] or any neonatal morbidity.

Pre-pregnancy obesity was defined as a body mass index (BMI) > 30 kg/m^2^ [29]. Gestational diabetes and normal glucose tolerance were confirmed according to the National Diabetes Data Group criteria [30]. In the diabetic cohort, HbA1c levels were monitored throughout the pregnancy.

### 4.1. Neurodevelopmental Assessment and ASD Diagnosis

ASD screening was performed using the Modified Checklist for Autism in Toddlers, Revised with Follow-Up (M-CHAT-R/F) [31]. Clinical diagnosis was established by a child psychiatrist using the Autism Diagnostic Observation Schedule, Second Edition (ADOS-2) as the gold standard observational instrument [32].

Additionally, neurodevelopment was assessed at 24–26 months of age using the Bayley Scales of Infant and Toddler Development, Third Edition (Bayley-III) [33]. All psychometric data were collected and analyzed by two specialists blinded to the study groups.

### 4.2. Collection and Storage of Samples and DNA Purification

Cord blood was collected immediately after birth in 3 mL K3-EDTA tubes in the delivery room and immediately stored at −80 °C until DNA extraction. DNA of cord blood samples was purified using the Blood Genomic Prep Mini Spin Kit (Cytiva Amersham™, Little Chalfont, UK). DNA concentrations were measured on the Qubit 3.0 fluorometer using the Qubit dsDNA HS Assay Kit (Thermo Fisher Scientific, Waltham, MA, USA).

### 4.3. Bisulfite Conversion and Methyl-Sequencing Procedures

We employed a sequencing-based method capable of covering over three million CpG sites. Bisulfite conversion of DNA and library preparations were performed using the TruSeq-Methyl-Capture-EPIC Library Prep Kit (Illumina Inc., San Diego, CA, USA) [34], which targets 3.34 million CpG sites, following the manufacturer’s recommendations. Library sizes were assessed with a 4200 TapeStation (Agilent Technologies, Santa Clara, CA, USA), and the concentration was determined with the Qubit dsDNA HS Assay Kit (Thermo Fisher Scientific, Waltham, MA, USA). Sequencing was conducted at the Instituto Tecnológico y de Energías Renovables (ITER, Santa Cruz de Tenerife, Canary Islands, Spain) using a NovaSeq 6000 Sequencing System (Illumina Inc., San Diego, CA, USA) with 110 bp paired-end, single-indexed reads along with 1% of a PhiX Control-V3 (Illumina Inc., San Diego, CA, USA).

### 4.4. Bioinformatics Analysis to Obtain Cytosine Methylation

We used bcl2fastq v2.20 [35] to perform sample demultiplexing and Bismark v0.24.0 [36] for bisulfite mapping and methylation calling against the GRCh37/hg19 [37] reference genome. The Bismark-based pipeline transformed the sequence and reads into fully bisulfite-converted forward (C → T) and reverse read (G → A) conversions of the forward strand versions. These transformed reads were then aligned to similarly converted versions of the reference genome (also C → T and G → A converted). The best alignment among the four possible bisulfite alignments was compared to the normal genomic sequence, and the methylation status of all cytosine positions in each read was inferred.

### 4.5. Methylation Analyses

Methylation analysis was conducted using the methylKit R package (version 1.32.1) [38]. Bases with coverage below 10× and above the 99.9th percentile of coverage in each sample were discarded to reduce potential biases associated with enrichment. The calculateDiffMeth function was used to identify CpG sites or regions with methylation differences using logistic regression models, including maternal age as a continuous covariate. Other variables, such as newborn sex, maternal race/ethnicity, and mode of delivery, were controlled by design through matching during cohort selection. Gestational age was excluded from the statistical models to prevent overfitting, given that it showed no significant baseline differences across the study groups. *p*-values were adjusted to q-values to control the false discovery rate (FDR) in multiple hypothesis testing using the Sliding Linear Model (SLIM). The MethSeg function from the genomation R package was applied to segment the methylome into regions with similar differential methylation profiles.

Differentially methylated (DM) CpGs were defined using a stringent > 25% threshold and q-value < 0.01—surpassing the common ≥10% cutoff—to ensure high-confidence results within our pilot cohort. Differentially methylated regions (DMRs), defined as clusters of ≥3 DM CpGs within ≤1000 base pairs, were conversely considered significant if differences were ≥10%, with a q-value < 0.01. DMRs were annotated according to their genomic coordinates (GRCh37/hg19). Genes containing one or more overlapping DMRs (DMR-associated genes) were identified to characterize the epigenetic landscape of our samples. Positive and negative methylation difference coefficients refer to hypermethylation and hypomethylation, respectively.

### 4.6. Gene Set Enrichment Analysis

Functional enrichment analysis of the DMR-associated genes was performed using the Gene Ontology Biological Process (GO:BP) annotation dataset through the web-based enrichment analysis tool of the Gene Ontology resource (https://geneontology.org/; accessed 15 January 2026), which implements the PANTHER overrepresentation test (Fisher’s exact test with false discovery rate correction) against the Homo sapiens reference gene list [12,39]. Statistical significance for the identified pathways was defined by a False Discovery Rate (FDR)-corrected q-value < 0.05.

Additionally, DMR-associated genes identified in both comparisons (ASD vs. IODM and ASD vs. IHM) were cross-referenced with the Simons Foundation Autism Research Initiative (SFARI) Gene database (accessed 15 January 2026; 2026 Q1 release comprising 1176 candidate genes) [13] to contextualize candidate loci according to established ASD risk categories: Category 1 (High Confidence), Category 2 (Strong Candidate), Category 3 (Suggestive Evidence), and Category S (Syndromic).

## 5. Conclusions

In conclusion, despite the inherent limitations of a pilot study, our findings contribute to the generation of the hypothesis that a panel of differentially methylated genes in cord blood could represent candidate loci for future evaluation as early markers of ASD risk. Furthermore, such a panel could include, among others, the *PCDHA1*–*PCDHA8* cluster, in which ASD-associated methylation differences have been identified for the first time. However, these results must be interpreted with caution, as they require validation in larger cohorts that include functional assessments.

## Figures and Tables

**Table 1:** Overlap between DMR-associated genes identified in both comparisons (ASD vs. IODM and ASD vs. IHM) and ASD candidate genes cataloged in the SFARI database [13]. ASD candidate genes concordant across both IODM and IHM comparisons are highlighted in bold (*n* = 29), with the *PCDH* gene cluster also highlighted in blue.

SFARI Category	ASD vs. IHM (*n* = 45)	ASD vs. IODM (*n* = 52)
1: High Confidence	*CACNA1A*, *CASZ1*, ***GABRB3***, *GRIN1*, ***MYT1L***, *NLGN2*, ***PCDH19***, *ZMYND8*	*ANKRD11*, *DEAF1*, *DMPK*, *EBF3*, ***GABRB3***, ***MYT1L***, ***PCDH19***
2: Strong Candidate	*ABAT*, *ABCA13*, ***AGAP2***, ***BCAS1***, ***CACNA1H***, ***CDH22***, ***CMIP***, ***CNTN4***, ***CSMD1***, *CUX1*, *CYFIP1*, ***DLGAP2***, *DOCK4*, ***GNAS***, ***KCNQ2***, *NOTCH1*, ***OXT***, *PBX1*, ***PCDHA1***–***PCDHA8***, *POLA2*, *PRKAR1B*, *PTPRC*, ***SAMD11***, *SNTG2*, *SRGAP3*, ***TRAPPC9***, ***ZMIZ1***	***AGAP2***, *ARHGEF10*, **BCAS1**, ***CACNA1H***, *CASKIN1*, ***CDH22***, ***CMIP***, ***CNTN4***, ***CSMD1***, *DIP2C*, *DLGAP1*, ***DLGAP2***, ***GNAS***, *GPC6*, *HIVEP3*, *HTR3A*, *KCNC1*, ***KCNQ2***, *KIF13B*, *MUC4*, ***OXT***, ***PCDHA1***–***PCDHA10***, ***SAMD11***, ***SNTG2***, *TPO*, ***TRAPPC9***, ***ZMIZ1***
3: Suggestive Evidence	***BCL11B***	***BCL11B***, *LMTK3*, *MSX2*
S: Syndromic	***HERC2***, ***NOVA2***	*GALNT2*, ***HERC2***, *MTSS2*, ***NOVA2***, *PACS2*, *TAF1*

## Data Availability

The processed DNA methylation datasets generated and analyzed during the current study using the methylKit R package are publicly available in the Zenodo repository at https://doi.org/10.5281/zenodo.20746328 (accessed on 18 June 2026).

## Supplementary Materials

The following supporting information can be downloaded at: https://www.mdpi.com/article/10.3390/ijms27188038/s1.
