# Supplementary material for: Targeted 3.34-Million CpG Site Sequencing Reveals Preliminary Cord Blood Epigenetic Alterations of Autism Spectrum Disorder: A Pilot Study Highlighting the PCDHA1–PCDHA8 Cluster
Source: Int J Mol Sci. 2026 Sep 9;27(18):8038. doi: 10.3390/ijms27188038 (PMC13607789; doi:10.3390/ijms27188038)
Supplement: Supplementary file 1 [file ijms-27-08038-s001.zip › Table S7.pdf]

Table S7. Gene Ontology (GO) [12] enrichment analysis of DMR-associated genes identified in the ASD group versus the IODM group.

Uniquely Mapped IDS: 20580, 567 out of 582, Unmapped IDs: 11, Multiple mapping information: 16.

Biological Process	Ref.	List	Expected	Fold Enrichment	P-value	FDR
**Homophilic cell adhesion**	175	21	4.9	4.24	2.61×10^{-8}	7.59×10^{-5}
Cell-cell adhesion	556	37	15.7	2.35	1.59×10^{-6}	2.56×10^{-3}
Cell adhesion	965	54	27.3	1.98	1.99×10^{-6}	2.89×10^{-3}
RTK signaling pathway	429	30	12.1	2.47	5.60×10^{-6}	6.26×10^{-3}
Enzyme-linked signaling	630	38	17.8	2.13	1.28×10^{-5}	1.09×10^{-2}
**Neuron development**	906	52	25.6	2.03	1.54×10^{-6}	2.81×10^{-3}
Cell development	2328	102	65.8	1.55	5.78×10^{-6}	5.60×10^{-3}
Cell differentiation	3775	146	106.8	1.37	4.33×10^{-5}	3.50×10^{-2}
Cellular development process	3778	146	106.8	1.37	4.37×10^{-5}	3.34×10^{-2}
Developmental process	5934	229	167.8	1.36	3.74×10^{-8}	9.05×10^{-5}
Anatomic structural development	5359	217	151.6	1.43	1.69×10^{-9}	1.23×10^{-5}
Neuron differentiation	1130	59	32.0	1.85	7.15×10^{-6}	6.49×10^{-3}
Generation of neurons	1204	62	34.1	1.82	5.71×10^{-6}	5.92×10^{-3}
Neurogenesis	1386	70	39.2	1.79	2.10×10^{-6}	2.78×10^{-3}
Nervous system dev.	2269	113	64.2	1.76	1.54×10^{-9}	2.24×10^{-5}
System development	3655	161	103.4	1.56	2.14×10^{-9}	1.04×10^{-5}
**Multicellular organism development**	4053	174	114.6	1.52	2.49×10^{-9}	9.04×10^{-6}
**Multicellular organism process**	6526	243	184.6	1.32	2.37×10^{-7}	4.92×10^{-4}

Ref., references number.
