# Supplementary material for: Targeted 3.34-Million CpG Site Sequencing Reveals Preliminary Cord Blood Epigenetic Alterations of Autism Spectrum Disorder: A Pilot Study Highlighting the PCDHA1–PCDHA8 Cluster
Source: Int J Mol Sci. 2026 Sep 9;27(18):8038. doi: 10.3390/ijms27188038 (PMC13607789; doi:10.3390/ijms27188038)
Supplement: Supplementary file 1 [file ijms-27-08038-s001.zip › Table S1.pdf]

Table S1. Maternal characteristics and perinatal outcomes, perinatal outcomes and Bayley test domain scores for infants included in the ASD group versus infants born of mothers with obesity and gestational diabetes, without ASD (IODM), and versus healthy infants of healthy mothers (IHM).

	**Infants with ASD** (*n* = 2)	**Control 1 IODM** (*n* = 6)	**Control 2 IHM** (*n* = 6)	**P_1**	**P_2**
**Maternal characteristics**					
Age (years)	38.5 ± 2.1	33.8 ± 5.6	33.2 ± 1.7	0.158	0.011
Body mass index (kg/m^2)	43.5 ± 4.4	37.6 ± 3.8	20.0 ± 1.9	0.120	<0.001
Parity = 0, n (%)	1 (50)	4 (67)	4 (67)	0.643	0.643
**Perinatal outcomes**					
Cesarean, n (%)	1 (50)	1 (17)	2 (33)	0.464	0.643
Gestational age (days)	267.5 ± 6.4	273.7 ± 6.3	275.0 ± 4.1	0.277	0.090
Newborn weight (g)	3905 ± 742	3789 ± 536	3339 ± 275	0.813	0.471
Weight percentile (%)	89.3 ± 15.0	78.9 ± 27.3	66.4 ± 23.2	0.637	0.249
LGA n (%)	1 (50)	4 (67)	-	0.643	0.250
Sex = Female, n (%)	1 (50)	3 (50)	3 (50)	-	-
Apgar test at 1st minute	9.0 ± 0.0	9.0 ± 0.0	9.0 ± 0.0	-	-
Apgar test at 5 minutes	9.0 ± 0.0	8.8 ± 0.4	9.0 ± 0.0	0.604	-
**Bayley test domain scores**					
Cognitive	72.5 ± 24.7	96.7 ± 4.9	124.5 ± 12.4	0.050	0.006
Language	59.5 ± 4.8	91.0 ± 19.1	124.7 ± 8.6	0.010	<0.001
Motor	74.5 ± 2.1	96.0 ± 9.6	117.0 ± 11.6	0.024	0.003
**Score < 95, n (%)**					
Cognitive	2 (100)	1 (17)	-	0.107	0.036
Language	2 (100)	5 (83)	-	1.000	0.036
Motor	2 (100)	3 (50)	-	0.464	0.036

Results are shown as percentages or mean and standard deviation. LGA, large for gestational age.

P_1: comparison between the ASD group and the IODM. P_2: comparison between ASD group and IHM. Data normality was assessed by inspecting the frequency distributions and the Kolmogorov–Smirnov test. Differences were studied using Student's t-test. Comparison between proportions was performed using the chi-squared test and the Fisher's exact test with small sample sizes (< 5). Statistical analyses were performed with IBM SPSS v.31.0 (Armonk, NY).
