# Supplementary material for: Targeted 3.34-Million CpG Site Sequencing Reveals Preliminary Cord Blood Epigenetic Alterations of Autism Spectrum Disorder: A Pilot Study Highlighting the PCDHA1–PCDHA8 Cluster
Source: Int J Mol Sci. 2026 Sep 9;27(18):8038. doi: 10.3390/ijms27188038 (PMC13607789; doi:10.3390/ijms27188038)
Supplement: Supplementary file 1 [file ijms-27-08038-s001.zip › Table S2.pdf]

Table S2. Differentially methylated region-associated genes identified in the ASD group compared to the IODM and IHM groups (n = 249).

ABCC13, ABCC4, ABHD17A, ADARB2, ADCY10P1, ADCY9, ADGRA2, AGAP2, AHRR, AIRN, ALPG, ALPI, ANKRD20A11P, APC2, ARHGAP26, ASB2, ATP11A, B4GALNT4, BAIAP2, BCAS1, BCL11B.

C2CD2L, C3orf84, C8orf31, C9orf139, CACNA1H, CAMSAP3, CARD9, CCDC57, CCKBR, CDH22, CDHR1, CDKL4, CHFR, CLDN15, CLIC3, CMIP, CNTN4, CNTNAP1, CPLANE1, CRTC1, CRYL1, CSMD1, CST7, CYP2D6, CYTH2, CZ1P-ASNS.

DCAF4L1, DENND3, DGCR8, DGKA, DIRAS3, DISP3, DLEU1, DLGAP2, DNAJC3, DOCK11, EFNA2, EHBP1L1, EPHA1-AS1, ESYT2, EXD3, FAM157A, FAM157B, FCGBP, FLT4, FOCAD, FZD1, FZD7.

GABRB3, GALNT9, GET4, GFI1, GFPT2, GLI2, GNAS, GNAS-AS1, GNG12-AS1, GNG7, GOLGA6L22, GPR39, GPRC5A, GREM2, GRIN2C, H3P4, HAGH, HCN2, HDAC10, HERC2, HERC2P10, HHIPL1, HOXA6, HOXA-AS3, HOXB3, HSF2BP, HYAL2, ICAM3, IGF1R, IGF2BP1, IGF2R, IGFBP7-AS1.

KALRN, KCNH3, KCNN1, KCNQ1, KCNQ2, KIF26B, KLC3, KLHL30, LAMA2, LINC00539, LINC00987, LINC01115, LINC01145, LINC01237, LINC01475, LINC01880, LINC02470, LINC02645, LIPC, LOC100132077, LOC100288728, LOC100420587, LOC101929829, LOC102723373, LOC105376360, LOC105376917, LOC171391, LOC728392, LRRC14, LRRC24, LYPD1.

MAEA, MAST1, MCF2L, MED16, MEG3, MIPEPP3, MRPS6, MYOM2, MYT1L, NBPF19, NDUFA10, NEURL1B, NFATC1, NFYA, NOVA2, NOX5, NR5A1, NTM, NTSR1, OR10AC1, OR2L13, OXT, PANX2, PAX8, PAX8-AS1, PCBP3, PCDH19, PCDHA1, PCDHA2, PCDHA3, PCDHA4, PCDHA5, PCDHA6, PCDHA7, PCDHA8, PDCD6-AHRR, PDE4DIPP4, PDE6B, PDLIM4, PEG13, PEMT, PKD1, PKP3, PLEC, PLEKHG5, PLIN3, PM20D1, PNPLA2, PPP1R13L, PPP1R16A, PPP1R2B, PRDM16, PRKAG2, PRKXP1, PRMT2, PTBP1, PTPRM, PTPRN2, PYY2.

RASSF4, RB1, RIMBP2, RIN2, RNF157, RPH3AL, RPTOR, RXFP3, S100A13, SAMD11, SCARA5, SDK2, SEMA5B, SEPTIN7P9, SH2B2, SHC2, SLC17A9, SLC2A1, SLC6A18, SNHG14, SNRPN, SNTG2, SNURF, SOGA3, SORT1, SPATA3-AS1, SPESP1, SRMS, SYNJ2, SYTL1.

TBCD, TCHH, TGIF1, TIMM23B, TIMM23B-AGAP6, TMCO3, TMSB15B, TNFRSF25, TRAP1, TRAPPC9, TRIM6, TRIM6-TRIM34, TSPAN9, USH1G, VIPR2, WDR25, WDR27, WDR97, WNT7B, WWC1, ZDHHC11B, ZDHHC14, ZFYVE28, ZMIZ1, ZNF135, ZNF335, ZNF469, ZNF837.
