# Supplementary material for: Targeted 3.34-Million CpG Site Sequencing Reveals Preliminary Cord Blood Epigenetic Alterations of Autism Spectrum Disorder: A Pilot Study Highlighting the PCDHA1–PCDHA8 Cluster
Source: Int J Mol Sci. 2026 Sep 9;27(18):8038. doi: 10.3390/ijms27188038 (PMC13607789; doi:10.3390/ijms27188038)
Supplement: Supplementary file 1 [file ijms-27-08038-s001.zip › Table S3.pdf]

Table S3. Gene Ontology [12] enrichment analysis (GO) of DMR-associated genes common to both comparisons (ASD vs. IODM and ASD vs. IHM). Uniquely Mapped IDS: 20580, 206 out of 213; Unmapped IDs, 43, Multiple mapping information, 8.

GO biological process completed	Reference	List	Expected	Fold enrich.	P-value	FDR
**Homophilic cell-cell adhesion**	175	15	1.8	8.28	4.34×10^{-10}	6.31×10^{-6}
Cell-cell adhesion	556	24	5.8	4.17	3.84×10^{-9}	1.86×10^{-5}
Cell adhesión	965	28	10.0	2.80	8.33×10^{-7}	1.73×10^{-3}
**Morphogenesis in neuron differentiation**	448	16	4.6	3.45	1.86×10^{-5}	2.08×10^{-2}
Neuron development	906	25	9.4	2.6	8.13×10^{-6}	1.18×10^{-2}
Developmental process	5934	91	61.4	1.48	1.76×10^{-5}	2.14×10^{-2}
Anatomical structure development	5359	87	55.5	1.57	2.88×10^{-6}	4.66×10^{-2}
Neuron differentiation	1130	30	11.7	2.57	2.04×10^{-6}	3.70×10^{-2}
Geneneration of neurons	1204	30	12.5	2.41	9.66×10^{-6}	1.28×10^{-2}
Neurogenesis	1386	32	14.3	2.23	2.16×10^{-5}	2.24×10^{-2}
Nervous system development	2269	54	23.5	2.30	3.42×10^{-9}	2.48×10^{-5}
System development	3655	73	37.8	1.93	6.67×10^{-9}	2.43×10^{-5}
Multicellular organism development	4053	77	42.0	1.84	1.76×10^{-8}	5.11×10^{-5}
Multicellular process	6526	103	67.5	1.52	3.81×10^{-7}	9.24×10^{-4}
Anatomical structure morphogenesis	2287	45	23.7	1.90	2.27×10^{-5}	2.20×10^{-2}
**Cell communication**	5314	83	55.0	1.51	2.62×10^{-5}	2.38×10^{-2}
**Signaling**	5270	82	54.5	1.50	3.54×10^{-5}	3.03×10^{-2}

Enrich, enrichment.
