# Supplementary material for: Targeted 3.34-Million CpG Site Sequencing Reveals Preliminary Cord Blood Epigenetic Alterations of Autism Spectrum Disorder: A Pilot Study Highlighting the PCDHA1–PCDHA8 Cluster
Source: Int J Mol Sci. 2026 Sep 9;27(18):8038. doi: 10.3390/ijms27188038 (PMC13607789; doi:10.3390/ijms27188038)
Supplement: Supplementary file 1 [file ijms-27-08038-s001.zip › Table S4.pdf]

Table S4. Complete list of DMR-associated genes overlapping with the SFARI Gene database, by comparison.

For each overlapping gene, all individual DMR loci detected in that comparison are listed separately (genomic coordinates, GRCh37/hg19), together with the corresponding FDR-corrected q-value and percentage methylation difference (positive = hypermethylation; negative = hypomethylation, relative to ASD infants). SFARI rank denotes the gene's overall rank in the SFARI database.

Gene	SFARI category	SFARI rank	Comparison	DMR locus (GRCh37/hg19)	FDR q-value	Meth. diff. (%)
ANKRD11	1 (High Confidence)	14	ASD vs. IODM	chr16:89408316-89408454	5.41e-03	-12.2%
CACNA1A (locus 1/2)	1 (High Confidence)	45	ASD vs. IHM	chr19:13319534-13319644	3.54e-03	+10.5%
CACNA1A (locus 2/2)	1 (High Confidence)	45	ASD vs. IHM	chr19:13409686-13409985	1.22e-05	-13.5%
CASZ1	1 (High Confidence)	182	ASD vs. IHM	chr1:10698344-10698573	1.86e-09	+10.9%
DEAF1	1 (High Confidence)	70	ASD vs. IODM	chr11:692353-693617	4.30e-08	+17.1%
DMPK	1 (High Confidence)	154	ASD vs. IODM	chr19:46274348-46274630	1.65e-06	-13.6%
EBF3	1 (High Confidence)	104	ASD vs. IODM	chr10:131669338-131669488	2.14e-11	-14.0%
GABRB3	1 (High Confidence)	35	ASD vs. IHM	chr15:26874076-26878880	4.13e-155	+11.8%
GABRB3	1 (High Confidence)	35	ASD vs. IODM	chr15:26874283-26874638	4.81e-04	+20.7%
GRIN1	1 (High Confidence)	80	ASD vs. IHM	chr9:140040199-140040486	8.91e-03	+12.9%
MYT1L	1 (High Confidence)	53	ASD vs. IHM	chr2:1846994-1852598	9.41e-34	-13.4%
MYT1L	1 (High Confidence)	53	ASD vs. IODM	chr2:1898572-1904749	4.22e-15	-12.3%
NLGN2	1 (High Confidence)	147	ASD vs. IHM	chr17:7311960-7318355	3.83e-08	-12.4%
PCDH19	1 (High Confidence)	25	ASD vs. IHM	chrX:99664457-99665231	5.09e-03	+12.1%
PCDH19	1 (High Confidence)	25	ASD vs. IODM	chrX:99665064-99665231	2.07e-06	+11.6%
ZMYND8	1 (High Confidence)	176	ASD vs. IHM	chr20:45947786-45962922	8.28e-06	-12.4%
ABAT	2 (Strong Candidate)	467	ASD vs. IHM	chr16:8779989-8807308	5.42e-03	-10.8%
ABCA13	2 (Strong Candidate)	361	ASD vs. IHM	chr7:48494569-48494729	8.14e-07	+11.0%
AGAP2	2 (Strong Candidate)	568	ASD vs. IHM	chr12:58131827-58132022	1.03e-45	-18.3%
AGAP2	2 (Strong Candidate)	568	ASD vs. IODM	chr12:58131815-58133411	8.84e-08	-18.2%
ARHGEF10	2 (Strong Candidate)	575	ASD vs. IODM	chr8:1845701-1849746	5.64e-04	-11.0%
BCAS1	2 (Strong Candidate)	666	ASD vs. IHM	chr20:52680912-52687016	2.90e-33	-13.1%
BCAS1	2 (Strong Candidate)	666	ASD vs. IODM	chr20:52680912-52687007	6.54e-49	-16.4%
CACNA1H	2 (Strong Candidate)	241	ASD vs. IHM	chr16:1208140-1208720	2.34e-07	-20.4%
CACNA1H	2 (Strong Candidate)	241	ASD vs. IODM	chr16:1208140-1208758	2.82e-12	-15.6%
CASKIN1	2 (Strong Candidate)	477	ASD vs. IODM	chr16:2229304-2229418	4.73e-22	-16.5%
CDH22	2 (Strong Candidate)	740	ASD vs. IHM	chr20:44838776-44839110	1.12e-04	-16.9%
CDH22	2 (Strong Candidate)	740	ASD vs. IODM	chr20:44829631-44829821	3.58e-18	-10.2%
CMIP	2 (Strong Candidate)	587	ASD vs. IHM	chr16:81615181-81617690	7.01e-08	+12.6%
CMIP	2 (Strong Candidate)	587	ASD vs. IODM	chr16:81616834-81617693	1.34e-06	+10.6%
CNTN4	2 (Strong Candidate)	249	ASD vs. IHM	chr3:3057232-3102906	3.16e-13	+11.0%
CNTN4	2 (Strong Candidate)	249	ASD vs. IODM	chr3:3057232-3102906	9.54e-21	+12.0%
CSMD1	2 (Strong Candidate)	271	ASD vs. IHM	chr8:3141765-3142187	9.98e-06	+13.2%
CSMD1	2 (Strong Candidate)	271	ASD vs. IODM	chr8:3102340-3142187	8.72e-11	+13.9%
CUX1	2 (Strong Candidate)	315	ASD vs. IHM	chr7:101596204-101596599	8.45e-07	+16.6%
CYFIP1	2 (Strong Candidate)	259	ASD vs. IHM	chr15:22915934-22916009	3.28e-04	-17.8%
DIP2C	2 (Strong Candidate)	524	ASD vs. IODM	chr10:530655-531148	6.43e-10	-17.8%
DLGAP1	2 (Strong Candidate)	333	ASD vs. IODM	chr18:3666034-3679453	3.46e-03	+11.2%
DLGAP2 (locus 1/2)	2 (Strong Candidate)	280	ASD vs. IHM	chr8:689872-691167	3.38e-04	+11.8%
DLGAP2 (locus 2/2)	2 (Strong Candidate)	280	ASD vs. IHM	chr8:1431217-1431300	9.28e-12	-14.2%
DLGAP2 (locus 1/3)	2 (Strong Candidate)	280	ASD vs. IODM	chr8:832366-832765	1.34e-03	+12.0%
DLGAP2 (locus 2/3)	2 (Strong Candidate)	280	ASD vs. IODM	chr8:1321261-1321765	2.78e-65	-17.3%
DLGAP2 (locus 3/3)	2 (Strong Candidate)	280	ASD vs. IODM	chr8:1440662-1440751	2.70e-09	-14.1%
DOCK4	2 (Strong Candidate)	368	ASD vs. IHM	chr7:111368367-111368588	2.04e-06	+15.2%
GNAS (locus 1/4)	2 (Strong Candidate)	444	ASD vs. IHM	chr20:57414835-57415237	4.89e-05	-10.2%
GNAS (locus 2/4)	2 (Strong Candidate)	444	ASD vs. IHM	chr20:57416888-57417018	5.96e-18	+29.1%
GNAS (locus 3/4)	2 (Strong Candidate)	444	ASD vs. IHM	chr20:57429919-57430015	1.69e-03	-12.2%
GNAS (locus 4/4)	2 (Strong Candidate)	444	ASD vs. IHM	chr20:57463455-57463700	1.17e-04	+10.1%
GNAS	2 (Strong Candidate)	444	ASD vs. IODM	chr20:57416857-57417070	7.74e-03	+19.1%
GPC6	2 (Strong Candidate)	487	ASD vs. IODM	chr13:95024759-95024900	5.58e-10	+10.4%
HIVEP3	2 (Strong Candidate)	445	ASD vs. IODM	chr1:42245564-42249204	3.52e-42	+13.9%
HTR3A	2 (Strong Candidate)	447	ASD vs. IODM	chr11:113846879-113848188	6.81e-04	+10.3%

Gene	SFARI category	SFARI rank	Comparison	DMR locus (GRCh37/hg19)	FDR q-value	Meth. diff. (%)
KCNC1	2 (Strong Candidate)	408	ASD vs. IODM	chr11:17758060-17758207	1.67e-18	+13.1%
KCNQ2 (locus 1/2)	2 (Strong Candidate)	235	ASD vs. IHM	chr20:62052119-62052342	1.18e-08	+12.1%
KCNQ2 (locus 2/2)	2 (Strong Candidate)	235	ASD vs. IHM	chr20:62084546-62084658	4.18e-05	-14.5%
KCNQ2 (locus 1/2)	2 (Strong Candidate)	235	ASD vs. IODM	chr20:62052119-62052888	6.24e-04	+17.6%
KCNQ2 (locus 2/2)	2 (Strong Candidate)	235	ASD vs. IODM	chr20:62084542-62084683	3.86e-26	-19.9%
KIF13B	2 (Strong Candidate)	760	ASD vs. IODM	chr8:28974382-28984419	3.01e-07	+20.8%
MUC4	2 (Strong Candidate)	454	ASD vs. IODM	chr3:195537567-195539200	2.08e-05	-11.3%
NOTCH1 (locus 1/2)	2 (Strong Candidate)	413	ASD vs. IHM	chr9:139406308-139406694	4.82e-06	-15.1%
NOTCH1 (locus 2/2)	2 (Strong Candidate)	413	ASD vs. IHM	chr9:139416102-139417394	5.00e-03	-15.1%
OXT	2 (Strong Candidate)	696	ASD vs. IHM	chr20:3052151-3052355	3.01e-04	-15.3%
OXT	2 (Strong Candidate)	696	ASD vs. IODM	chr20:3052115-3052412	1.31e-43	-10.2%
PBX1	2 (Strong Candidate)	497	ASD vs. IHM	chr1:164545635-164545686	6.57e-03	-16.1%
PCDHA1 (locus 1/2)	2 (Strong Candidate)	778	ASD vs. IHM	chr5:140175994-140176055	5.89e-04	-12.6%
PCDHA1 (locus 2/2)	2 (Strong Candidate)	778	ASD vs. IHM	chr5:140222271-140222351	4.30e-04	-10.3%
PCDHA1	2 (Strong Candidate)	778	ASD vs. IODM	chr5:140242290-140242639	4.59e-05	-14.7%
PCDHA10	2 (Strong Candidate)	626	ASD vs. IODM	chr5:140242290-140242639	4.59e-05	-14.7%
PCDHA2 (locus 1/2)	2 (Strong Candidate)	701	ASD vs. IHM	chr5:140175994-140176055	5.89e-04	-12.6%
PCDHA2 (locus 2/2)	2 (Strong Candidate)	701	ASD vs. IHM	chr5:140222271-140222351	4.30e-04	-10.3%
PCDHA2	2 (Strong Candidate)	701	ASD vs. IODM	chr5:140242290-140242639	4.59e-05	-14.7%
PCDHA3	2 (Strong Candidate)	702	ASD vs. IHM	chr5:140222271-140222351	4.30e-04	-10.3%
PCDHA3	2 (Strong Candidate)	702	ASD vs. IODM	chr5:140242290-140242639	4.59e-05	-14.7%
PCDHA4	2 (Strong Candidate)	628	ASD vs. IHM	chr5:140222271-140222351	4.30e-04	-10.3%
PCDHA4	2 (Strong Candidate)	628	ASD vs. IODM	chr5:140242290-140242639	4.59e-05	-14.7%
PCDHA5	2 (Strong Candidate)	545	ASD vs. IHM	chr5:140222271-140222351	4.30e-04	-10.3%
PCDHA5	2 (Strong Candidate)	545	ASD vs. IODM	chr5:140242290-140242639	4.59e-05	-14.7%
PCDHA6	2 (Strong Candidate)	779	ASD vs. IHM	chr5:140222271-140222351	4.30e-04	-10.3%
PCDHA6	2 (Strong Candidate)	779	ASD vs. IODM	chr5:140242290-140242639	4.59e-05	-14.7%
PCDHA7	2 (Strong Candidate)	546	ASD vs. IHM	chr5:140222271-140222351	4.30e-04	-10.3%
PCDHA7	2 (Strong Candidate)	546	ASD vs. IODM	chr5:140242290-140242639	4.59e-05	-14.7%
PCDHA8	2 (Strong Candidate)	703	ASD vs. IHM	chr5:140222271-140222351	4.30e-04	-10.3%
PCDHA8	2 (Strong Candidate)	703	ASD vs. IODM	chr5:140242290-140242639	4.59e-05	-14.7%
PCDHA9	2 (Strong Candidate)	780	ASD vs. IODM	chr5:140242290-140242639	4.59e-05	-14.7%
POLA2	2 (Strong Candidate)	547	ASD vs. IHM	chr11:65059158-65061401	7.68e-04	+12.9%
PRKAR1B	2 (Strong Candidate)	549	ASD vs. IHM	chr7:750980-751336	4.64e-07	-11.0%
PTPRC	2 (Strong Candidate)	380	ASD vs. IHM	chr1:198648702-198648962	4.39e-05	+10.1%
SAMD11	2 (Strong Candidate)	792	ASD vs. IHM	chr1:866937-874672	7.72e-103	-16.1%
SAMD11	2 (Strong Candidate)	792	ASD vs. IODM	chr1:861631-871620	4.22e-105	-16.3%
SNTG2	2 (Strong Candidate)	554	ASD vs. IHM	chr2:1357410-1377603	3.89e-13	+10.3%
SNTG2	2 (Strong Candidate)	554	ASD vs. IODM	chr2:1129547-1129964	1.45e-68	-16.6%
SRGAP3	2 (Strong Candidate)	648	ASD vs. IHM	chr3:9177692-9177975	9.99e-06	-14.1%
TPO (locus 1/2)	2 (Strong Candidate)	939	ASD vs. IODM	chr2:1481076-1481136	1.30e-04	+13.0%
TPO (locus 2/2)	2 (Strong Candidate)	939	ASD vs. IODM	chr2:1481214-1481412	1.34e-12	-11.1%
TRAPPC9 (locus 1/2)	2 (Strong Candidate)	256	ASD vs. IHM	chr8:141109686-141109969	6.34e-07	+14.3%
TRAPPC9 (locus 2/2)	2 (Strong Candidate)	256	ASD vs. IHM	chr8:141224959-141229527	1.28e-06	+12.0%
TRAPPC9 (locus 1/2)	2 (Strong Candidate)	256	ASD vs. IODM	chr8:141108662-141108938	4.68e-05	+14.1%
TRAPPC9 (locus 2/2)	2 (Strong Candidate)	256	ASD vs. IODM	chr8:141109703-141110033	2.05e-07	+12.7%
ZMIZ1	2 (Strong Candidate)	387	ASD vs. IHM	chr10:81034064-81034408	2.11e-03	+10.3%
ZMIZ1 (locus 1/2)	2 (Strong Candidate)	387	ASD vs. IODM	chr10:80999771-80999888	6.60e-59	-13.2%
ZMIZ1 (locus 2/2)	2 (Strong Candidate)	387	ASD vs. IODM	chr10:81034064-81034408	2.53e-11	+10.1%
BCL11B	3 (Suggestive Evidence)	980	ASD vs. IHM	chr14:99702577-99707182	2.40e-13	-10.8%
BCL11B	3 (Suggestive Evidence)	980	ASD vs. IODM	chr14:99701423-99707182	8.30e-03	-10.2%
LMTK3	3 (Suggestive Evidence)	993	ASD vs. IODM	chr19:49000424-49004474	2.40e-63	-13.5%
MSX2	3 (Suggestive Evidence)	1067	ASD vs. IODM	chr5:174152469-174156296	5.93e-13	+10.4%
GALNT2	S (Syndromic)	1142	ASD vs. IODM	chr1:230289702-230296979	4.19e-06	+11.2%
HERC2	S (Syndromic)	1111	ASD vs. IHM	chr15:28413667-28419660	4.64e-07	-11.8%
HERC2	S (Syndromic)	1111	ASD vs. IODM	chr15:28413667-28419745	3.51e-08	-10.2%
MTSS2	S (Syndromic)	1153	ASD vs. IODM	chr16:70713592-70714003	1.38e-12	-11.1%
NOVA2	S (Syndromic)	1166	ASD vs. IHM	chr19:46443831-46444149	4.71e-13	-13.6%

Gene	SFARI category	SFARI rank	Comparison	DMR locus (GRCh37/hg19)	FDR q-value	Meth. diff. (%)
NOVA2	S (Syndromic)	1166	ASD vs. IODM	chr19:46438660-46438845	3.28e-11	-12.4%
PACS2	S (Syndromic)	1114	ASD vs. IODM	chr14:105860904-105864430	5.54e-11	+13.5%
TAF1	S (Syndromic)	1092	ASD vs. IODM	chrX:70586159-70586655	3.67e-04	-11.5%
