# Supplementary material for: Targeted 3.34-Million CpG Site Sequencing Reveals Preliminary Cord Blood Epigenetic Alterations of Autism Spectrum Disorder: A Pilot Study Highlighting the PCDHA1–PCDHA8 Cluster
Source: Int J Mol Sci. 2026 Sep 9;27(18):8038. doi: 10.3390/ijms27188038 (PMC13607789; doi:10.3390/ijms27188038)
Supplement: Supplementary file 1 [file ijms-27-08038-s001.zip › Table S5.pdf]

Table S5. Gene Ontology (GO) [12] enrichment analysis of all 516 DMR-associated genes identified in the ASD vs IHM comparison. Uniquely mapped IDs: 20,580; 415 out of 430 mapped; unmapped IDs: 100; multiple mapping: 14.

Neurodevelopment-related pathways

Biological Process	Ref.	List	Expected	Fold enrichment	P-value	FDR
**Positive Regulation of excitatory postsynaptic potential**	33	6	0.69	8.72	5.44×10^{-5}	3.04×10^{-2}
Regulation signal transduction	3192	98	66.54	1.47	6.38×10^{-5}	3.44×10^{-2}
Modulation excitatory postsynaptic potential	53	8	1.10	7.24	1.30×10^{-5}	1.18×10^{-2}
Regulatory nervous systen process	131	13	2.73	4.76	3.66×10^{-6}	4.44×10^{-3}
**Homophilic cell-cell adhesion**	175	18	3.65	4.93	2.89×10^{-8}	7.01×10^{-5}
Cell-cell adhesion	556	32	11.59	2.76	2.50×10^{-7}	4.55×10^{-4}
Cell adhesion	965	41	20.12	2.04	1.50×10^{-5}	1.28×10^{-2}
Cellular process	15672	360	327.4	1.10	1.23×10^{-4}	4.84×10^{-2}
**Axon guidance**	232	15	4.84	3.10	1.14×10^{-4}	4.74×10^{-2}
Neuron projection guidance	234	15	4.8	3.07	1.29×10^{-4}	4.93×10^{-2}
Neuron development	906	40	18.89	2.12	7.71×10^{-6}	8.62×10^{-3}
Cell development	2328	76	48.53	1.57	7.62×10^{-5}	3.82×10^{-2}
Neuron differentiation	1130	50	23.56	2.12	4.99×10^{-7}	7.25×10^{-4}
Generation of neurons	1204	52	25.10	2.07	6.31×10^{-7}	8.34×10^{-4}
Neurogenesis	1386	58	28.89	2.01	4.22×10^{-7}	6.82×10^{-4}
Nervous system development.	2269	98	47.30	2.07	1.43×10^{-12}	2.08×10^{-8}
Cell morphologic neuron differentiation	448	23	9.3	2.46	7.67×10^{-5}	3.72×10^{-2}
**Chemical synaptic transmission**	424	22	8.84	2.49	9.08×10^{-5}	4.26×10^{-2}
Anterograde trans-synaptic signalign	424	22	8.84	2.49	9.08×10^{-5}	4.13×10^{-2}
Trans-synaptic signaling	453	23	9.44	2.44	8.75×10^{-5}	4.24×10^{-2}
Signaling	5270	147	109.86	1.34	5.44×10^{-5}	3.16×10^{-2}
Cell communication	5314	151	110.77	1.36	1.63×10^{-5}	1.31×10^{-2}
**Central Nervous System development**	1026	41	21.39	1.92	6.90×10^{-5}	3.58×10^{-2}
