# Supplementary material for: Targeted 3.34-Million CpG Site Sequencing Reveals Preliminary Cord Blood Epigenetic Alterations of Autism Spectrum Disorder: A Pilot Study Highlighting the PCDHA1–PCDHA8 Cluster
Source: Int J Mol Sci. 2026 Sep 9;27(18):8038. doi: 10.3390/ijms27188038 (PMC13607789; doi:10.3390/ijms27188038)
Supplement: Supplementary file 1 [file ijms-27-08038-s001.zip › Table S6.pdf]

Table S6. Gene Ontology (GO) [12] enrichment analysis of all 516 DMR-associated genes identified in the ASD vs IHM comparison. Uniquely mapped IDs: 20,580; 415 out of 430 mapped; unmapped IDs: 100; multiple mapping: 14.

Biological Process	Ref.	List	Expected	Fold Enrichment	P-value	FDR
**Metanephric tubule morphogenesis**	10	4	0.21	19.19	3.54×10^{-5}	2.45×10^{-2}
Anatomical structure development	5359	166	111.71	1.49	7.91×10^{-9}	2.88×10^{-5}
Developmental process	5934	174	123.70	1.41	1.51×10^{-7}	3.14×10^{-4}
Anatomical structure morphogenesis	2287	76	47.67	1.59	3.53×10^{-5}	2.56×10^{-2}
Multicellular organism development	4053	143	84.49	1.69	1.96×10^{-11}	9.48×10^{-8}
Multicellular organismal process	6526	191	136.04	1.40	2.24×10^{-8}	6.52×10^{-5}
Metanephric tubule development	20	5	0.42	11.99	4.60×10^{-5}	2.79×10^{-2}
Metanephric epithelium development	23	5	0.48	10.43	9.49×10^{-5}	4.18×10^{-2}
System development	3655	135	76.19	1.77	4.17×10^{-12}	3.03×10^{-8}
**Metanephric nephron morphogenesis**	18	5	0.38	13.33	2.63×10^{-5}	2.01×10^{-2}
Metanephros morphogenesis	25	6	0.52	11.51	1.00×10^{-5}	1.04×10^{-2}
**Positive regulation of excitatory postsynaptic potential**	33	6	0.69	8.72	5.44×10^{-5}	3.04×10^{-2}
Regulation of signal transduction	3192	98	66.54	1.47	6.38×10^{-5}	3.44×10^{-2}
Modulation of excitatory postsynaptic potential	53	8	1.10	7.24	1.30×10^{-5}	1.18×10^{-2}
Regulation of nervous system process	131	13	2.73	4.76	3.66×10^{-6}	4.44×10^{-3}
**Homophilic cell-cell adhesion**	175	18	3.65	4.93	2.89×10^{-8}	7.01×10^{-5}
Cell-cell adhesion	556	32	11.59	2.76	2.50×10^{-7}	4.55×10^{-4}
Cell adhesion	965	41	20.12	2.04	1.50×10^{-5}	1.28×10^{-2}
**Axon guidance**	232	15	4.84	3.10	1.14×10^{-4}	4.74×10^{-2}
Neuron development	906	40	18.89	2.12	7.71×10^{-6}	8.62×10^{-3}
Cell development	2328	76	48.53	1.57	7.62×10^{-5}	3.82×10^{-2}
Neuron differentiation	1130	50	23.56	2.12	4.99×10^{-7}	7.25×10^{-4}
Generation of neurons	1204	52	25.10	2.07	6.31×10^{-7}	8.34×10^{-4}
Neurogenesis	1386	58	28.89	2.01	4.22×10^{-7}	6.82×10^{-4}
Nervous system development	2269	98	47.30	2.07	1.43×10^{-12}	2.08×10^{-8}
**Chemical synaptic transmission**	424	22	8.84	2.49	9.08×10^{-5}	4.26×10^{-2}
Anterograde trans-synaptic signaling	424	22	8.84	2.49	9.08×10^{-5}	4.13×10^{-2}
Trans-synaptic signaling	453	23	9.44	2.44	8.75×10^{-5}	4.24×10^{-2}
Signaling	5270	147	109.86	1.34	5.44×10^{-5}	3.16×10^{-2}
Cell communication	5314	151	110.77	1.36	1.63×10^{-5}	1.31×10^{-2}
**Positive regulation of cell differentiation**	857	37	17.86	2.07	3.84×10^{-5}	2.53×10^{-2}
Regulation of developmental process	2527	82	52.68	1.56	3.93×10^{-5}	2.49×10^{-2}
Regulation of cell differentiation	1576	59	32.85	1.80	1.26×10^{-5}	1.23×10^{-2}
**Central nervous system development**	1026	41	21.39	1.92	6.90×10^{-5}	3.58×10^{-2}
**Regulation of multicellular organismal development**	1428	52	29.77	1.75	1.03×10^{-4}	4.40×10^{-2}

Ref., references number.
